# Complexity of MRI induced heating on metallic leads: Experimental measurements of 374 configurations

**DOI:** 10.1186/1475-925X-7-11

**Published:** 2008-03-03

**Authors:** Eugenio Mattei, Michele Triventi, Giovanni Calcagnini, Federica Censi, Wolfgang Kainz, Gonzalo Mendoza, Howard I Bassen, Pietro Bartolini

**Affiliations:** 1 Dept. of Technologies and Health, Italian National Institute of Health, Roma, Italy; 2 Center for Devices and Radiological Health, Food and Drug Administration, Rockville, MD, USA

## Abstract

**Background:**

MRI induced heating on PM leads is a very complex issue. The widely varying results described in literature suggest that there are many factors that influence the degree of heating and that not always are adequately addressed by existing testing methods.

**Methods:**

We present a wide database of experimental measurements of the heating of metallic wires and PM leads in a 1.5 T RF coil. The aim of these measurements is to systematically quantify the contribution of some potential factors involved in the MRI induced heating: the length and the geometric structure of the lead; the implant location within the body and the lead path; the shape of the phantom used to simulate the human trunk and its relative position inside the RF coil.

**Results:**

We found that the several factors are the primary influence on heating at the tip. Closer locations of the leads to the edge of the phantom and to the edge of the coil produce maximum heating. The lead length is the other crucial factor, whereas the implant area does not seem to have a major role in the induced temperature increase. Also the lead structure and the geometry of the phantom revealed to be elements that can significantly modify the amount of heating.

**Conclusion:**

Our findings highlight the factors that have significant effects on MRI induced heating of implanted wires and leads. These factors must be taken into account by those who plan to study or model MRI heating of implants. Also our data should help those who wish to develop guidelines for defining safe medical implants for MRI patients. In addition, our database of the entire set of measurements can help those who wish to validate their numerical models of implants that may be exposed to MRI systems.

## Background

Magnetic resonance imaging (MRI) has become the imaging procedure of choice for extensive clinical evaluation and diagnosis. Unlike conventional radiography and computed tomographic imaging, which make use of potentially harmful radiation (X-rays), MRI has many advantages, including its no ionizing nature and the unparallel ability to discriminate different soft tissues without contrast media. However, the substantial benefits of MRI are often denied to patients known to have implanted medical devices such as pacemakers (PM) and implantable cardioverter devices (ICD). The outstanding increases in both MRI usage and cardiac device-based therapy have resulted in an estimated 50–75% probability of a patient being indicated for an MRI over the lifetime of their device [1,2].

MRI induced heating is one of the known risks for implanted PM and ICD: the RF field energy generated during MRI procedures may be coupled into conductive leads in two major ways [3]:

1) The conductive lead acts as an antenna capable of receiving and supporting the frequency of the MRI unit's RF field (63.8 MHz for 1.5 Tesla systems or 127.6 MHz for 3.0 Tesla systems). This mechanism can create the resonant waves.

2) The implant acts as an electrical "short circuit" to the electrical potentials induced within the body by the MRI RF field.

Each of these effects induces a RF current which flows from the lead into the surrounding tissues. As a consequence of the high electrical resistance of these tissues, resistive heating is then generated at the lead-tissue interface, causing a temperature increase that could be dangerous for the patients.

The scientific and medical literature clearly shows that the MRI-induced heating on metallic leads cannot be immediately neglected: Sommer et al. [4] demonstrated the potential for heating as much as 23.5°C at specific absorption rate (SAR) levels of only 1.3 W/kg in a 0.5 Tesla MRI unit. Achenbach et al. [5] reported pacing lead tip temperature elevation in leads not attached to an implanted pulse generator (IPG) of >63°C during 90 seconds of MRI. Roguin [6] observed in an animal model a lose capture for 12 hours following an MRI, concluding that "... some edema occurred at the tip-lead tissue interface, which subsequently resolved." Martin and Coman [7] observed 9.4% of their patients undergo "significant" changes to their pacing thresholds after MRI. Konings et al. [8] reported heating around intravascular guide wires by resonating RF waves of 26°C to 74°C after 30 seconds. Bassen et al. [9] measured a temperature rise of about 0.5°C (local SAR = 320 W/kg) for a stent exposed to the RF field of a 1.5 T MRI birdcage coil at 64 MHz; using the same experimental set-up, a temperature increase of 8.6°C was observed at the bare end of an insulated 24-cm long wire (local SAR = 5680 W/kg). Rezai et al. [10] reported deep brain stimulator temperature elevations at the electrode tip of >25°C within 15 minutes of MR imaging. This data is particularly disturbing when one realizes that thermal ablation procedures are typically performed at temperatures of approximately 50–60°C. At the same time, over 300 patients to date have been scanned without any significant clinical difficulty or complication [11].

Such widely varying results indicate how the performance of an implanted or interventional device undergoing MRI is a very complex problem. While it is relatively easy to demonstrate a heating or induced voltage problem, it is far more difficult to prove a solution to these problems, due to the complex and unpredictable nature of the MRI interaction. Several factors influence the degree of heating: the whole body specific absorption rate, the patient position in the coil, the type of imaging sequence, the patient characteristics, the duration of imaging procedure, the body structure being imaged, the type and position of transmit coil, the lead design, the lead orientation within the patient, the degree of perfusion near the device, the temperature measurement procedure, the respiratory phase, etc. Many of these parameters are currently either not recognized or inadequately addressed by existing testing methods [3]. In a previous study we investigated the variability in temperature and SAR measurements using fluoroptic^® ^temperature probes on the tip of a PM lead (ref). In this paper we present a wide database of experimental measurements of the heating of metallic wires and PM leads in a 1.5 T RF coil. The aim of these measurements is to systematically quantify the contribution of some potential factors involved in the MRI induced heating: the length and the geometric structure of the lead; the implant location within the body and the lead path; the shape of the phantom used to simulate the human trunk and its relative position inside the RF coil. In addition, the data collected will serve as an open-access experimental database for numerical models validation.

## Methods

### Exposure System

**Figure 1:**
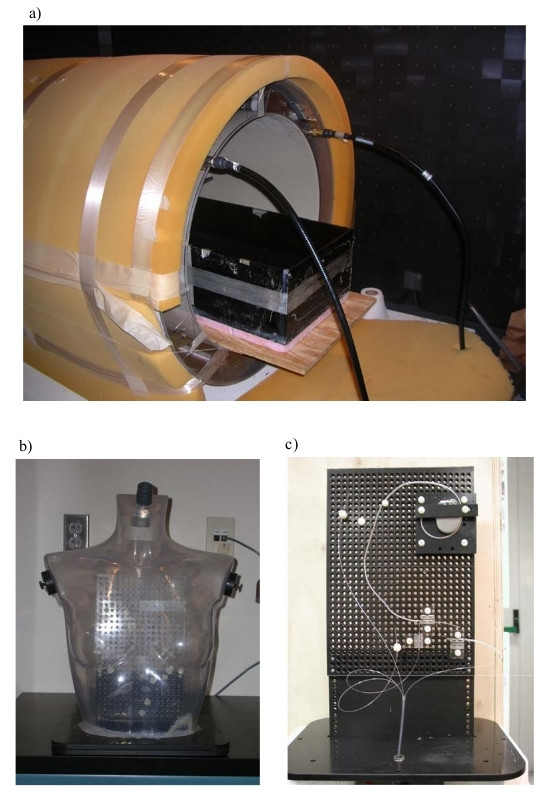
**RF coil and phantoms**. a) MRI birdcage coil with rectangular box phantom inside coil; b) PVC grid placed inside the phantom to support the pacemaker implant and the fluoroptic^® ^temperature probes; c) Human-shaped phantom filled with HEC gel.

We performed temperature measurements at the Center for Device and Radiological Health (CDRH), Food and Drug Administration (FDA) in Rockville, MD, USA. We used a full-size RF coil (length 113 cm, inner diameter 62 cm) with 16 legs forming the classic Birdcage configuration (Figure 1a). Tuning capacitors are placed on each of the legs, resulting in a low-pass structure. This system is the same as those used in 1.5 T clinical systems. The coil was fed by a quadrature power divider so to produce a circularly polarized B1 fields. The birdcage coil was housed in an anechoic chamber and the exposure was realized by a RF amplifier that delivers over 130 Watts at 64 MHz.

**Figure 2:**
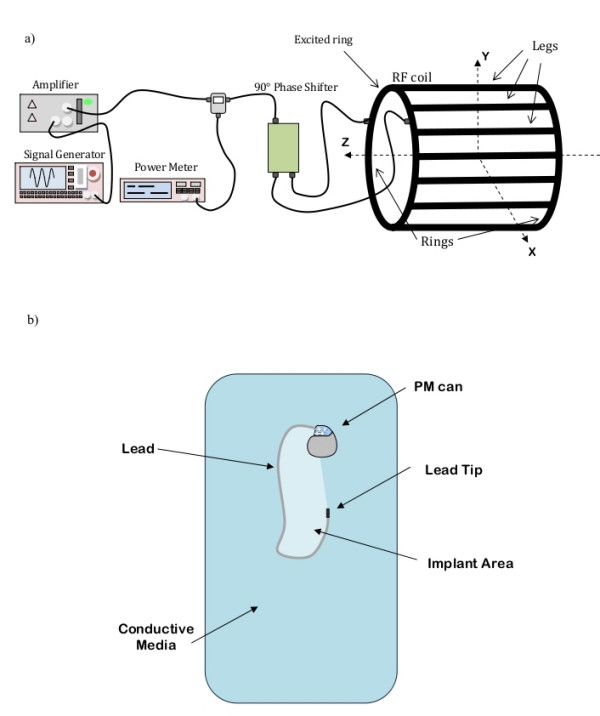
**Experimental set-up and terminology**. a) Experimental set-up and MRI coil configuration terminology; b) Sketch and terminology for a PM implants.

A schematic representation of the experimental set-up used is shown in Figure 2a.

### Phantoms

We used two geometries as human trunk simulator: a rectangular plastic box (Figure 1b) and a PVC human-shaped phantom (Figure 1c). Both phantoms were filled with a saline gel composed of Hydroethylcellulose gelling agent (HEC) and NaCl to meet the general requirements of the ASTM F2182-02a standard for testing of MRI heating of implants [12]. The mixture we used produced conductivity at 64 MHz of about 0.7 Sm-1, corresponding to a salinity of about 0.4% by weight, and permittivity of 79. The amount chosen for the HEC (2% by weight) allowed implants to be placed in the gel, moved and replaced, but, at the same time, provided a barrier to rapid thermal convection. Any art voids that are created by movements of implants filled in less than 20 minutes.

The rectangular box phantom (35 × 61 × 19 cm deep) was filled to a depth of 11.5 cm with the gel. The dimensions conformed to the ASTM 2182 standard [12], except for the width of our box. This was narrower than the ASTM specifications to allow the phantom to fit in the RF coil (62 cm inner diameter). The total amount of gel was 24.6 l, corresponding to a weight of 24.7 kg. The box was surrounded with 2.6 cm of rigid foam for thermal insulation and it was placed on a wood board about 10 cm far from the bottom of the RF coil (Figure 1a). This foam allowed us to perform calorimetry studies to determinate the whole body SAR induced in the phantom. A 26 × 18 cm grid was submerged in the gel to support the implants and maintained a consistent separation distance between the metallic structures, the phantom gel surface and the temperature probes. The grid was adjusted so that the top of the implant was positioned 2 cm below the phantom surface, which resulted in a final position of the grid about 10 cm down the centre of the coil, along the vertical transversal axis (y axis – Figure 2a).

The human-shaped trunk simulator was designed and built at the Dept. of Technology and Health of the National Institute of Health in Rome. The simulator consists of a torso-shaped transparent PVC phantom of the size of a 45 kg female and of an internal volume of 25 liter. The pacemaker and its leads were fixed on a PVC grid placed inside the phantom (Figure 1b).

Both the rectangular box and the human-shaped phantom were placed in various positions inside the RF coil: in this way, we could compare the heating induced on the same implant configurations, but with the phantom differently positioned respect to the coil. The MRI coil configuration and axes are shown in Figure 2a. The terminology for implants is illustrated in Figure 2b.

### Implants

The measurements we performed aimed at the identification of the major contributes involved in the heat generation: (1) the position of the implant inside the phantom, (2) the length of the wire and, the thickness of the insulation sheath, (3) the wire geometries (shape) and (4) its position of the implant including its lead with respect to the RF coil. In the following pages, we use the term "*configuration*" (config.) to refer to a single experimental set-up; for each configuration we measured the temperature increase and we estimated the local SAR. The proposed configurations were properly modified from one another in order to investigate the specific contributes to the heating of the four factors previously mentioned. Inside the rectangular box phantom we first simulated the implant with conductive wires (radius 0.5 mm) of various shapes and length; we tested both bare and insulated wires (insulation thickness 0.5 mm and 1 mm). For the insulated wires, the exposed tip length was 1 mm at only one end of the implant. then we replaced the metallic wires with a dual chamber PM (Elect D, *Sorin Biomedica CRM*, Italy) with its 62 cm-long leads. We tested both unipolar and bipolar leads (mod. S80T and S80TB, *Sorin Biomedica CRM*, Italy), either attached or not to the PM can (metallic case).

**Figure 3:**
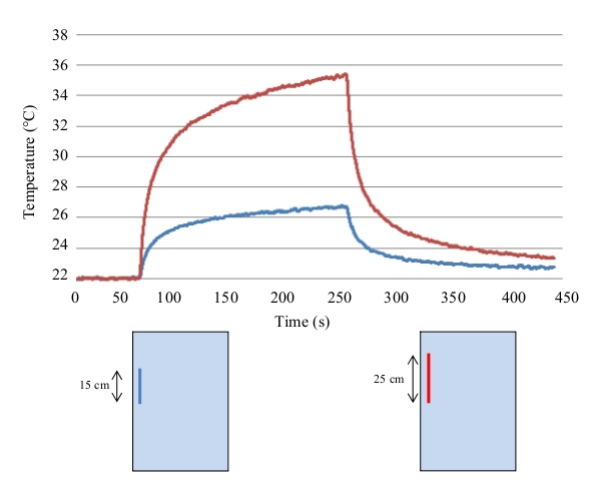
**Temperature increase at the lead tip**. Example of temperature increase versus time curves measured with fluoroptic^® ^probes at the tip of the implant, during the experiments inside the RF coil. The two lines refers to the configurations reported below.

In a real PM implant, since the length of the lead may not fit the patient's anatomy and size, the excess length may be wrapped near or around the PM can. As a consequence, the length of the lead and the area covered by the implant can significantly vary from patient to patient, thus creating many configurations of implants. In addition, some papers on MRI safety state that a higher heating is induced at the tips of implants which cover a large area [4,10]. From this point, we dedicated a group of measurements to investigate the effect of the implant area, which is the area delimited by the lead, the PM can and the line connecting the lead tip to the PM center of mass (Figure 3b). In the experiments with metallic wires, we compare the temperature increase for wires of different length but with the same geometry (i.e. same shape) (24 config.) When using a real PM with its lead, we tested several configurations without changing the position of the PM can, whereas the total area of the implant was varied by wrapping the exceeding lead near the PM body or by changing the lead geometry (75 config.). Also the presence of the PM can respect to the unattached lead was investigated (18 config.).

Frequency resonance phenomena in various kinds of linear metallic leads and wires undergoing MRI procedures (e.g. catheters used in interventional radiology) have been hypothesized by several groups [13-15]. Resonance is said to occur when the lead is an integral fraction of a half wavelength [15]. The wavelength of 64 MHz is 4.68 m in air but is almost 12 times smaller in saline (due to the dielectric constant and conductibility of saline). Yeung et al [15] illustrates that a 25 cm wire length is close to the resonant length for 64 MHz. We investigated the contribution of resonance to the heating of the lead length by measuring the temperature rise at the tip of three straight wires of different lengths: 15 cm, 25 cm 40 cm (15 config.)

The implants used in our measurements were placed inside a material with an electrical conductivity greater than zero; thus, the presence of an insulation sheath and its thickness may affect the amount of heating induced at the implant tip by concentrating the "exiting" current from the wire in the saline. We tested both bare wires and wires insulated with thermoplastic sheaths of two thicknesses (0.5 mm and 1 mm) (12 conf).

In clinical practice, the PM chassis may be typically located in the left or right pectoral region of the patient, with a lead path that significantly changes in the two cases. Our interest was to understand if one implant location may be considered safer than the other, concerning the heating induced by the MRI exposure. To this aim, we shaped the metallic wires in order to simulate the two typical implants paths for a PM lead, corresponding to a left and right pectoral implant position. We compared the temperature increase in the two cases (70 config.). Left and right pectoral implants were also studied when using a real PM with its lead (32 conf). In addition, we reproduced several implant configurations, both with metallic wires and real PM leads, which do not correspond to a physiological implant condition, but which could give interesting result in order to understand how the geometry of the implant may affect the amount of the induced heating (114 config.).

With regard to heating of pacemaker leads it has been discussed whether imaging of brain and abdomen may pose potentially less risk than imaging of the thorax [16]. We tried to study this issue performing a separate group of measures where we moved our phantoms along the main axis of the RF birdcage coil, so to simulate MRI scanning of different organs (48 config.).

For most of the implant structures tested, we compared the temperature increases resulting for the same configurations but placed in different regions of the phantom. We studied this for metallic wires and with actual PM leads. In most of the previous studies published on MRI safety and compatibility with metallic implants, temperature measurements are performed inside human trunk simulators of quite simple geometry (box phantoms, rectangular phantoms with rectangular head). In order to evaluate whether the phantom's shape and geometry is a factor that could affect the amount of heating induced by MRI procedures, we placed a PM lead with its can on the left and right sides of a human-shaped phantom, so to reproduce actual left and right pectoral implants: in this study we used bipolar leads, and the lead paths were chosen to mimic a physiological implant condition (atrial and ventricular stimulation). The importance of the geometry of the phantom was studied by comparing the temperature increases resulting from the same configurations tested first inside the rectangular phantom, then in the human-shaped phantom (38 conf). The two phantoms had comparable total volume and were exposed to the same whole body SAR.

In all our experiments we measured the temperature increase and SAR at the tip of a "standard wire" (18-cm long, diameter 1 mm, insulated by thermoplastic tubing with a wall thickness of 0.5 mm)., this wire was always placed in the same location and position inside the phantoms: in this way we had a parameter to evaluate the reproducibility of our measurements. We also used the same wire to create a map of the temperature increase and SAR deposition inside the rectangular phantom, by positioning it in the four corners of the simulator (8 config.). We placed the wires 2 cm far from the edges of the box, orientated both along the Z and Y axis of the coil (a picture of the wire position can be found in the Additional file 1).

### Instrumentation

Temperature measurements were performed using a fluoroptic^® ^thermometer (Luxtron model 3100) with four separate model SMM probes. These plastic fiber probes (1 mm diameter) minimize perturbations of RF fields. The Luxtron system was operated at 8 samples per second, with resolution of 0.1°C. The background noise was in the range of Luxtron resolution. The terminal portion of the temperature probes were placed in transversal contact (i.e. the probe is perpendicular to the body of the lead-wire axis) with the lead tip: this contact configuration was demonstrated to minimize the measurements error associated to the physical dimensions of the probes. Temperature was also sampled on the PM can and, when using a bipolar lead, on the ring. Data acquisition was performed using the analog output of the Luxtron thermometer, an A/D converter and a 16-bit acquisition card (National Instrument, DAQCard-AI-16XE-50), installed on a standard notebook computer (Acer 1670, Windows XP OS) In previous studies [9,17] the positions and mounting of the temperature probes yielding the minimum error for temperature and SAR measurements were investigated. The best positioning is obtained when the Luxtron probe (actually the center of the pigmented part) in direct contact with the lead tip or the ring electrode. In this configuration the Luxtron probe and the lead tip or ring electrode are perpendicular to each other. Care was taken to ensure contact of the sensitive region of the Luxtron probe (center of the pigmented end of the Luxtron probe) and the lead tip or ring electrode. In such conditions the underestimations associated to SAR and temperature measurements are lower than 20%, and 7%, respectively.

### Exposure protocols

Each configuration tested inside the RF coil was characterized by a temperature versus time behavior made up of three phases: an initial base signal with no excitation in the coil was acquired for a period of 60 s, followed by a 200 s of exposure and a 200 s cooling phase. As a preliminary study, we performed calorimetry with the rectangular box phantom filled with saline (without gelling agent) to measure the whole body average SAR delivered to the phantom. This allowed us to determine the total power absorbed by the phantom (whole body SAR) [9]. The power supplied to the coil was about 55 W, producing inside the phantom a whole body average SAR of about 1 W/kg. Determining the whole-body SAR would allow us to extrapolate the data we collected in our lab on the heating of PM leads to values that could be expected to occur in a phantom placed in a clinical MRI system. For each temperature increase acquired, we also calculated the local SAR following the method described in IEEE C95.3-2002 [18]. This method leads to uncertainties of about ± 1–2 dB in the local SAR evaluation. Local SAR at the lead tip was calculated according to the definition of SAR by multiplying the initial slope of the temperature rise (dT/dt) with the specific heat capacity of the gel [9,19]. We used a slope-determining algorithm to properly select the linear portion of the initial temperature rise. The starting point was defined as the first sharp temperature increase from baseline; the initial linear slope was estimated on the number of samples which maximized the Pearson coefficient (R-squared) of the regression (range 25–50 samples).

## Results

We produced a database of 374 experimental measurement results from our investigation of several parameters affecting MR-induced heating. Results are grouped as a function of the parameter investigated (e.g., position of the implant inside the phantom, area covered by the implant, etc). A total of 58 configurations of implants are reported in this paper. The whole set of measurement data as well as the raw temperature data are provided as Excel sheets in the Additional file 1 (temperature measurements on metallic wires) and Additional file 2 (temperature measurements on PM leads). Significant temperature increases (relative to patient safety) were observed only at the lead tip, while no significant heating occurred either at the PM can or at the ring of bipolar leads. Therefore we report only the temperature increase at the lead tip. This increase is the absolute difference between temperature immediately before RF exposure and at the end of 200 seconds of exposure. An example of the temperature rise is shown in Figure 3.

### Position of the implant inside the phantom

**Figure 4:**
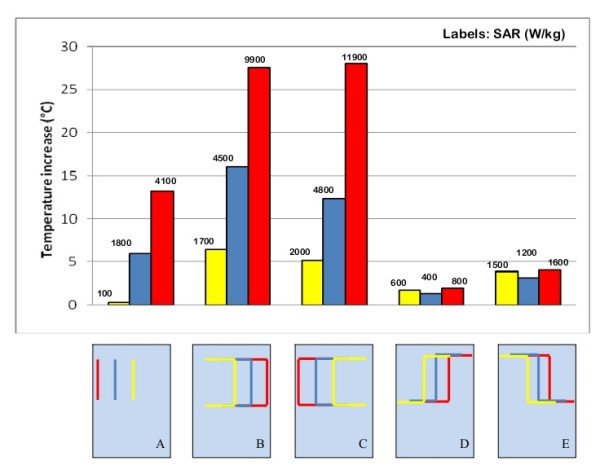
**Position of the implant inside the phantom**. Temperature increases and SAR measured at the tip of metallic wires placed in different positions inside the phantom. The uncertainty in the reported SAR value is ± 2 dB. Each group of bars refers to the implant configuration reported below.

We studied an insulated, straight wire that was 25-cm long and parallel with axis of coil (radius 0.5 mm, insulation thickness 0.5 mm, exposed bare tip length = 1 mm). This wire was placed in the middle of the rectangular box phantom. It produced no significant heating at its tip, whereas the temperature increase measured on the same wire placed close to the edge of the torso simulator was as high as 13°C (Figure 4A). The effect of the distance from the edge of the phantom is even more marked for longer wires with a complex path. When shaped to simulate a left implant position, with the main straight segment near one side of the phantom, the induced heating saturated the dynamic range of our of the A/D converter (temperature increase > 30°C). If moved to the central region of the torso simulator, the temperate increase was less than 8°C (Figure 4B–C). The same behavior was as well observed for metallic wire of different shape (Figure 4D–E) and for PM implants, regardless the particular path designed by the lead.

The local SAR values are consistent with the temperature increases: the highest power deposition is always reached in close proximity of the phantom edges.

### Length of straight wire implants

**Figure 5:**
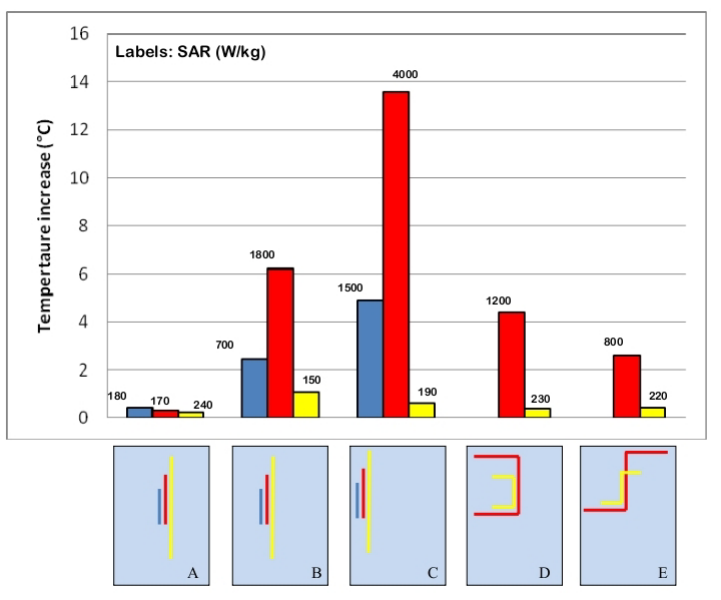
**Length of wire implants**. Temperature increases and SAR measured at the tip of metallic wires of different lengths (15 cm, 25 cm and 40 cm – A, B, C; 25 cm and 50 cm – D, E). The uncertainty in the reported SAR value is ± 2 dB. Each group of bars refers to the implant configuration reported below.

Inside the rectangular trunk simulator, we measured the temperature increases at the tip of three straight metallic wires of different length (15 cm 25 cm and 40 cm). Different locations were tested (Figure 5A–B–C). All the three wires had 0.5 mm-thick thermoplastic insulation, except for the tip (bare for 1 mm). The highest temperature increase (13.5°C) was obtained for the 25-cm long wire (Figure 5A–B–C). The induced heating was much less for the other two implants (4.3°C and 0.8°C for the 15 cm-long and 40 cm-long wire, respectively).

The SAR value are substantially consistent with the temperature increases; the highest SAR was reached at the tip of the 25 cm long wire, when placed close to the border of the rectangular phantom Figure 5C). The 15 cm and 40 cm wires, located in the same position, gave a significant lower SAR.

When the temperature increase are very small (<1°C), the SAR calculation with the method described in the previous section cannot be so accurate and leads to values not so significant.

### Area enclosed by the implant

**Figure 6:**
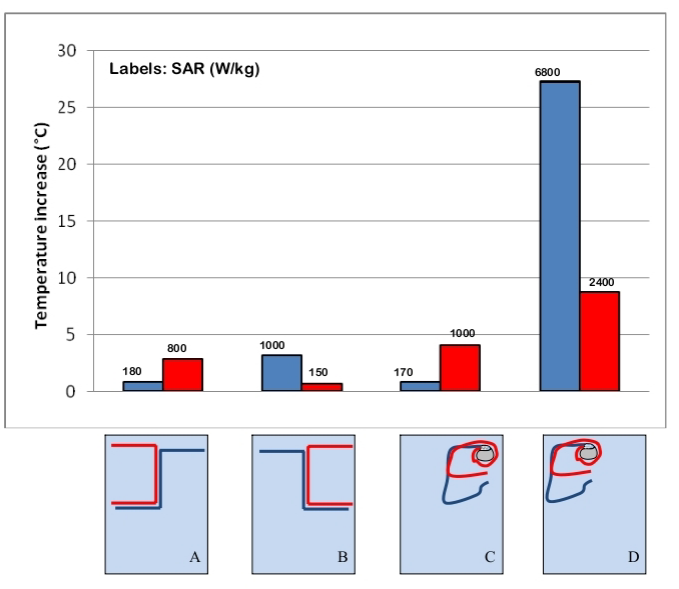
**Shaped and area enclosed by the implant**. Comparison between the temperature increases and SAR measured at the tip of metallic wires of different shape (A, B); comparison between the temperature increases and SAR for PM implants of different area, in different regions of the phantom. The uncertainty in the reported SAR value is ± 2 dB. Each group of bars refers to the implant configuration reported below.

We observed that a leads that formed a square shape and therefore had a large "loop area" (Figure 5D) did not always imply a higher heating. The same lead shape for a smaller implant area obtained by shaping a 25-cm long wire may produce a higher temperature increase than a 50-cm long wire with similar shaping, if placed more peripherally inside the phantom (see the Additional file 1 for details). Only when the main straight segments of the wires are located at the same distance from the phantom edges, the implant that covers a larger area produces a higher temperature increase at its tip (Figure 5D–E). In the same way, a no-loop PM implant (i.e. an implant with the lead that is not wrapped around the PM can) covered an area almost double than the one of a one-loop configuration (the lead makes one loop around the PM can): the former caused a temperature increase significantly higher (> 15°C) than the latter when both of the PM leads are placed near the border of the rectangular box simulator (Figure 6D). After moving both implants in the central region of the phantom, the major heating was obtained for the one-loop configuration (4°C versus 1°C of the no-loop implant; Figure 6C). Such results show that more than the area, the actual contribute to the heating is the length of the segments of the implant places next to the edge of the phantom. A similar behavior was as well observed by comparing the SAR values: the highest deposited power is reached at the tip of those implants that have long straight segment close to the borders of the phantom.

### Geometry and structure of the lead

Experimental measurements of metallic wires in phantoms showed significant differences between left and right pectoral implants. In particular, with the exposed tip in the left region of the rectangular phantom (in the physiological position of the heart ventricles), the right-implant wire, that is the wire shaped to simulate the lead path from the right pectoral region to the heart ventricle, produced a temperature increase of 2.6°C whereas for the left-implant wire (shaped to reproduce the lead path from the left pectoral region to the heart ventricle) we measured a temperature increase barely higher than 1°C (Figure 6A). An opposite configuration, with the tip of the two wires in the right region of the box simulator, produced opposite results: the highest heating was obtained for the left-implant wire (temperature increase of 3°C), whereas the temperature at the right-implant wire rose of less than 1°C (Figure 6B).

Inside the human-shaped phantom we reproduced left and right pectoral implants of the pacemaker can, with the lead paths chosen to most closely approximate a physiological configuration for a dual chamber PM, simulating implantation in both atrium and ventricle. In agreement with the measurements on the rectangular box phantom, we found that right-pectoral implants produced a higher heating that left-pectoral implants, in most of the configurations we studied. We also investigated if the different structures of unipolar and bipolar PM leads may affect the amount of heating caused by MRI exposure. We compared temperature increases with the each of two leads attached separately to the same PM can. The leads were located in various regions inside the rectangular phantom, in order to see if this would produce different degrees of heating at their tips.

**Figure 7:**
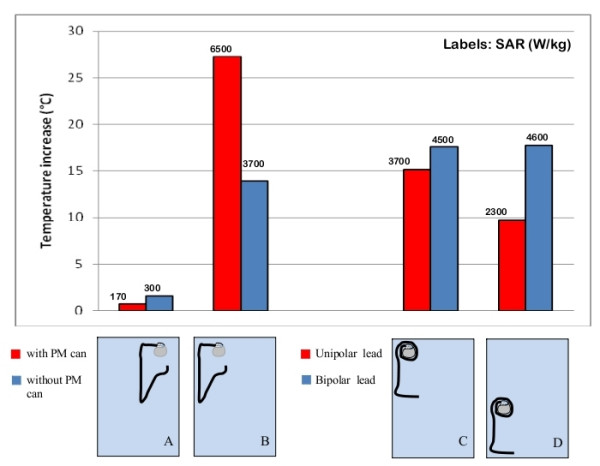
**Implant and lead structure**. Effect of the presence of the PM can on the temperature increase and SAR at the lead tip (A, B); different amounts of heating and SAR observed for unipolar and bipolar PM leads (C, D). The uncertainty in the reported SAR value is ± 2 dB. Each group of bars refers to the implant configuration reported below.

We found that for locations in the phantom where low temperature increases occurred, the behavior of the two types of leads is quite similar, whereas for locations in the phantom where heating was higher, the temperature increase for bipolar leads was significantly greater than for unipolar leads. A maximum difference of 8.1°C was observed, for one-loop PM configurations and with the leads placed close the edges of the phantom (Figure 7C–D).

In addition, we performed a group of measurements in order to evaluate how the presence of the PM can modifies the temperature increase at the lead tip. By comparing the results on PM leads that were either attached or unattached to a PM case, we observed a significant difference in the two situations. The presence of the PM can may either lead to an increase or decrease in the induced heating, depending on its position inside the phantom (Figure 7A–B). The same differences we found in the temperature increases, for the implant geometries and structures tested were as well found for the computed SAR values.

### Position of the phantom and the implant with respect to the RF coil

Several implant configurations were tested with the phantom placed at first in the center of the coil and then moved along the main coil axis towards the RF excited (driven) ring (Figure 1a) or the non-excited ring end. We found that the amount of heating strongly depends on the phantom position, regardless the phantom shape (either rectangular or human-shaped): the highest temperature increase was measured when the implant was located inside the central field area of the RF coil (± 13 cm far from the center along X, Y and Z axes – isocenter): since both metallic wires and PM leads were generally placed in the upper middle half region of the phantoms, closer to excited ring, the maximum heating was obtained when moving the phantom towards the non excited ring. As the implant was shifted out from the central field region of the coil, the temperature increase was less than it when the implant was placed in the isocenter. At a distance of about 30 cm from the isocenter, we observed a 10–30% temperature rise decrease, with respect to the one measured inside the central field area, depending on the particular implant configuration.

We previously observed that an implant located next to any of the edges of the phantom produces the highest temperature increase. Our interest was also to understand if the contribution to the heating was due to the fact that the implant was positioned near the edges of the phantom or near the leg of the RF coil. To this aim, we compared temperature measurements performed on implants located in the same position respect to the coil, but in different areas of the phantom (i.e. the phantom was moved but the implant was in a fixed location in the coil). A marked decrease in the heating was always observed when implants were moved from the edges to the center of the phantom, even without changing their positions respect to the RF coil.

In addition, the presence of the tuning capacitors in the low-pass birdcage coil may cause a local increase in the E field. However, this high field regions are rather small, and may additionally contribute to the lead heating only when both the phantom and the implant are positioned very close to the inner side of the coil.

Also with regard to the SAR, the position of the phantom inside the RF coil showed to be an important factor. For a typical PM implant configuration (no-loop, left implant), the deposited power goes from 2217.5 W/kg with the phantom moved towards the excited ring, to 4432.9 W/kg with the phantom moved towards the non excited ring (see Additional file 2 for further details).

### Geometry of the phantom

**Figure 8:**
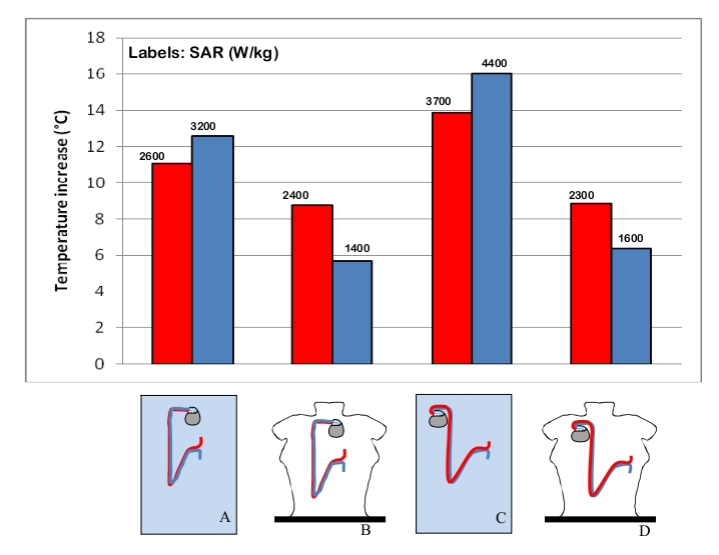
**Geometry of the phantom**. Effect of the phantom geometry on the induced heating and SAR for the same implant configuration reproduced inside the rectangular box phantom (A, C) and the human shaped phantom (B, D). The uncertainty in the reported SAR value is ± 2 dB. Each group of bars refers to the implant configuration reported below.

We measured induced heating for the same wire and lead configurations when implanted in the rectangular box and in the human-shaped phantom. Even though great care was taken in order to maintain the same distance between the implants and the borders of the phantom, we observed that, for almost all the implants we tested, the temperature increase measured inside the human-shaped phantom was about 6°C lower than in the rectangular trunk simulator (Figure 8). The lower temperature increases measured inside the human shaped phantom go with lower SAR value, compared to same configurations reproduced inside the rectangular phantom.

### Thickness of the insulation sheath

**Figure 9:**
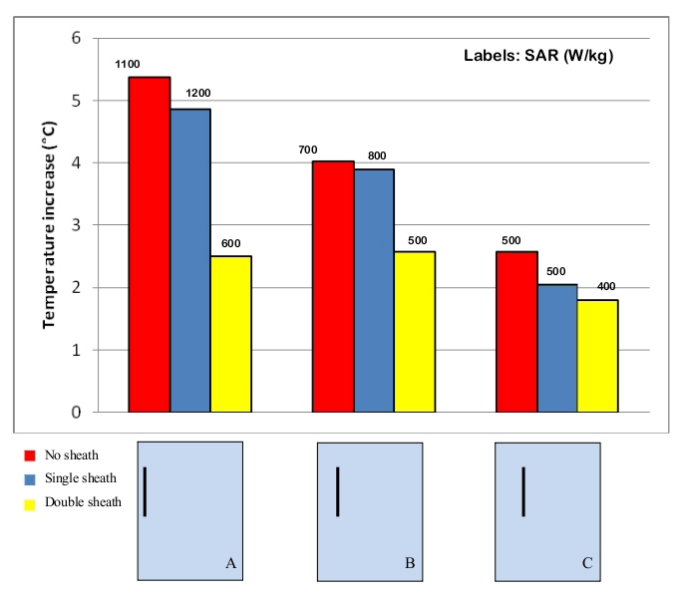
**Thickness of the insulation sheath**. Influence of the presence and thickness of the insulation sheath on the amount of the induced heating and SAR at the tip of a 15 cm long straight wire. The uncertainty in the reported SAR value is ± 2 dB. Each group of bars refers to the implant configuration reported below.

We compared the temperature increases for three straight wires of the same length (15 cm) with no insulation (bare wire), 0.5 mm thick insulation (single-sheath) and 1 mm thick insulation (double-sheath wire) thermoplastic sheaths. Different locations inside the rectangular phantom were tested (close to the edge, halfway between the edge and the center and in the center – Figure 9); the phantom was kept in the central field region of the coil, at the same distance from the coil legs and rings. We found that the bare and the single-sheath wire produced almost the same temperature increase, even if the bare wire reaches the steady state more slowly than the insulated wire. The heating at the tip of the double-sheath wire was instead significantly lower: a temperature increase of 2.2°C was measured, compared to 5.2°C of the bare and single-sheath wires. We also used a 25 cm-long bare wire to reproduce more realistic paths for a PM lead: several positions and geometries were tested and no significant heating was observed at the lead tip, even when the same configuration for an insulated wire (0.5 mm insulation thickness) had produced temperature increases greater than 10°C. The different dynamic in temperature rise leads to a SAR value that is higher for the insulated wire than for the bare one, even if the temperature increase is higher for the latter than the former.

## Discussion

Previous studies investigating MRI induced PM lead heating reported a large variability in the induced temperature increases versus the location of the lead in phantom and the location of the phantom within the coil. We performed extensive measurements inside a RF birdcage coil on metallic wires and PM leads, in order to identify the major contributors to heating, which may explain such variability in results. We collected a wide archive of experimental data consisting of 374 configurations, which reveal how complex the problem is and how many factors have to be taken into account in order to understand and model the heating-induction phenomenon. These factors include the position of the implant inside the phantom, the structure and the geometry of the lead, the thickness of the external insulation on the lead the presence of the PM can, the shape and size of the phantom, and the position of the phantom inside the RF coil.

Our data show clearly that the several factors are the primary influence on heating at the tip. Closer locations of the leads to the edge of the phantom and to the edge of the coil produce maximum heating. This is due to the electric field that is highest at the edges (radial and at the ends) of the phantom nearest the inner wall of the RF coil; in addition, the low-pass structure of the coil may additionally contribute to the heating if the implant is close to the tuning capacitors placed along the legs. The other factor is the length of the lead. Others have stated that the magnetic field is believed to be a primary contributor to heating of the lead. Several papers in the literature [4,10] chose a configuration of the pacemaker lead in the coronal plane to achieve a maximal magnetic induction area (larger loop producing larger area (area as shown in Figure 2b). This was done by others to maximize the heating at the lead tip. We found that the temperature increase is not always proportional to this area. Similar areas might give different temperature increases; in some cases we even observed a higher temperature increase on the tip of the implant with the smaller area, but with the lead located closer to the phantom's edges. We demonstrated that rather than the area, the length of the straight segments of the lead and their position inside the phantom and the coil determines the amount of heating.

The comparison between left and right implant configurations, both with metallic wires and PM leads, confirms that the area covered by the implant is not a crucial element in MRI induced heating. Right implants covered an area significantly smaller than the left counterparts and the temperature increases are higher for the former than the latter ones.

The influence of the insulation sheath on the MRI induced heating is still a widely discussed issue. The thickness of the silicone or polyurethane sheath of a PM lead (less than a millimeter) is very small compared to the wavelength (5 m) of the RF field used inside a MRI system. This would let us suppose that the presence of the insulation is not an important aspect to be considered in modeling the RF currents induced in the lead [20]. Actually, our experimental measurements prove that the amount of heating depends greatly on whether the metallic wire is provided with insulation or not. In addition, the thickness of the insulation seems to play an important role. For a bare wire that was shaped to reproduce the typical path of a PM lead, the RF-induced temperature increase was much lower than for the same configuration with an insulated wire of the same shape.

However, when comparing temperature increases on short straight wires (15 cm long) exposed under identical conditions, the higher heating is observed for the bare wire, compared to insulated wire of the same length and shape. For the bare, short wire the temperature reached after long exposures (steady state) is high, but the initial rate of rise (SAR at the wire tip) is lower. Furthermore, for short straight wires, the heating seems to decrease as the thickness of the insulation sheath increases.

The contribution of the lead structure (unipolar or bipolar) to the heating is strongly affected by the positioning of the lead itself inside the phantom: bipolar leads showed higher temperature increases than unipolar when placed close to the edge of the phantom; when placed in the central regions, heating was slightly higher for unipolar. Since this comparison was based on a limited number of positions and lead paths, no generalization or interpretation of these results can be attempted.

Clinical MRI birdcage coils are designed to have a homogenous RF magnetic field over the entire body of a patient. We did studies to mimic MRI imaging of a part of the body different from the thorax, such as the head or the abdomen, for a pacemaker patient. The phantom was shifted along the main (Z) axis of the RF coil. For these conditions, the highest heating is obtained with an implant that crosses the isocenter of the coil. As the distance of the center of the implant from the isocenter increases, the heating gets lower. Particularly, a marked decrease in temperature occurs when part of the implant is shifted along the Z axis of the coil out from the ring of the RF coil.

Temperature and SAR measurements we performed showed also the importance of the morphology of the phantom: the heating induced in the same implant configurations when placed in the rectangular phantom was higher than in the human-shaped phantom. The irregular surface of the human-shaped phantom implies a distance of the implant from the edges that varies point to point. This may partially explain such differences. Furthermore, the currents that are induced in the rectangular phantom, follow straight regular paths that provide a better coupling with straight metallic wires or leads. Our findings highlight the factors that have significant effects on RF-induced heating of implanted wires and leads. These factors must be taken into account by those who plan to study, model MRI heating of implants. Also our data should help those who wish to develop guidelines for defining safe medical implants for MRI patients. In addition, our database of the entire set of measurements can help those who wish to validate their numerical models of implants that may be exposed to MRI systems.

## Conclusion

Our study demonstrates how complex the MRI-induced heating phenomenon of pacemaker leads is and how many aspects are involved in this process. Experimental measurements inside a RF birdcage coil at 64 MHz revealed two major factors affecting the amount of lead tip heating: the length of the straight segments of the lead and their position inside the phantom. In particular, in order to have the worst case condition (i.e. maximize the induced heating and local SAR at the tip) inside a simulated torso, the following are necessary. The lead has to be placed parallel and next to the lateral edges of the phantom (parallel to the long axis of the phantom). The phantom has to be placed close to the inner wall of the RF coil, The length of the lead has to be close to the theoretical resonance length, at the frequency of the applied RF field (i.e. a half wavelength in the dielectric tissue medium). In addition the path of the lead has to be chosen as to obtain the best coupling with the E field distribution inside the phantom. With regards to the field generated by a birdcage RF coil typically used in most MRI scanners, the lead path which goes from the right pectoral region to the heart ventricle is the one that produces the best coupling with the E field. Other aspects play also an important role in MRI induced heating: the structure of the lead, the presence of the PM can, the geometry (shape) of the phantom and its position inside the coil. All these elements make it very time-consuming and expensive to perform extensive and exhaustive experimental measurements, as well as to develop accurate numerical models. Thus, while our presented data cannot be exhaustive, to our knowledge, they represent the largest database of MRI induced heating on thin metallic implants and pacemaker leads. The data are also intended to be used to validate numerical models.

## Disclaimer

The mention of commercial products, their sources, or their use in connection with material reported herein is not to be construed as either an actual or implied endorsement of such products by the U.S. Food and Drug Administration. The opinions expressed by of the author are not those of the US Food and Drug administration.

## Competing interests

The author(s) declare that they have no competing interests.

## Authors' contributions

EM carried out the experimental measures and drafted the manuscript. MT realized the human shaped-phantom and participated in the acquisition of the experimental data. GC has made substantial contributions to conception and interpretation of data. FC participated in the design of the experimental set-up, interpretation of data and revised the manuscript. WK participated in the design and realization of the exposure system and discussed the methodological issues related. GM helped in the preparation of the gel and in setting up and calibrating the temperature acquisition system. HB revised the final manuscript and gave the final approval for publication. PB gave the final approval for publication.

## Supplementary Material

Additional file 1 **Temperature and SAR measures on metallic wires**. The Excel file (2007 version) contains the temperature raw data collected during the RF exposure of metallic wires. For each configuration, which corresponds to a single column of the file, a schematic representation of the experimental set-up is reported. For each configuration, we also calculated the difference between the baseline and the highest temperature reached during the RF exposure (dT – °C), as well as the SAR (W/kg), from the slope of the initial temperature increase (see Methods section for details). The file is divided into 5 sheets, which refers to different shapes of the metallic wire: - straight wires; - left imp. wires (insulated wires shape to simulated the PM lead path from the left pectoral region to the heart ventricle); - right imp. wires (insulated wires shape to simulated the PM lead path from the right pectoral region to the heart ventricle); - bare wires; - left and right imp. wires (left imp. wires and right imp. wires simultaneously exposed to the RF signal). The first column of each sheet contains the time reference for the temperature values. Click here for file

Additional file 2 **Temperature and SAR measures on PM leads**. The Excel file (2007 version) contains the temperature raw data collected during the RF exposure of PM leads. For each configuration, which corresponds to a single column of the file, a schematic representation of the experimental set-up is reported. For each configuration, we also calculated the difference between the baseline and the highest temperature reached during the RF exposure (dT - °C), as well as the SAR (W/kg), from the slope of the initial temperature increase (see Methods section for details). The file is divided into 5 sheets, which refers to different locations or structures of the lead: - left imp.-0,1,2 loops (PM can positioned in the upper left region of the phantom, with the lead that makes 0, 1 or 2 loops around it); - right imp.-0,1,2 loops (PM can positioned in the upper right region of the phantom, with the lead that makes 0, 1 or 2 loops around it); - Bipolar leads; - rect. VS human-shaped phantom (comparison between same configuration in the rectangular and in the human-shaped phantom). The first column of each sheet contains the time reference for the temperature values. Click here for file
